# Dynamic links between theta executive functions and alpha storage buffers in auditory and visual working memory

**DOI:** 10.1111/j.1460-9568.2010.07217.x

**Published:** 2010-05

**Authors:** Masahiro Kawasaki, Keiichi Kitajo, Yoko Yamaguchi

**Affiliations:** 1 Rhythm-based Brain Computation Unit, RIKEN BSI-TOYOTA Collaboration Center2-1, Hirosawa, Wako-shi, Saitama 351-0198, Japan; 2 Laboratory for Dynamics of Emergent Intelligence, RIKEN Brain Science InstituteSaitama, Japan; 3 PRESTO, Japan Science and Technology Agency (JST)Saitama, Japan

**Keywords:** alpha, brain oscillations, phase synchronization, theta, working memory

## Abstract

Working memory (WM) tasks require not only distinct functions such as a storage buffer and central executive functions, but also coordination among these functions. Neuroimaging studies have revealed the contributions of different brain regions to different functional roles in WM tasks; however, little is known about the neural mechanism governing their coordination. Electroencephalographic (EEG) rhythms, especially theta and alpha, are known to appear over distributed brain regions during WM tasks, but the rhythms associated with task-relevant regional coupling have not been obtained thus far. In this study, we conducted time–frequency analyses for EEG data in WM tasks that include manipulation periods and memory storage buffer periods. We used both auditory WM tasks and visual WM tasks. The results successfully demonstrated function-specific EEG activities. The frontal theta amplitudes increased during the manipulation periods of both tasks. The alpha amplitudes increased during not only the manipulation but also the maintenance periods in the temporal area for the auditory WM and the parietal area for the visual WM. The phase synchronization analyses indicated that, under the relevant task conditions, the temporal and parietal regions show enhanced phase synchronization in the theta bands with the frontal region, whereas phase synchronization between theta and alpha is significantly enhanced only within the individual areas. Our results suggest that WM task-relevant brain regions are coordinated by distant theta synchronization for central executive functions, by local alpha synchronization for the memory storage buffer, and by theta–alpha coupling for inter-functional integration.

## Introduction

Working memory (WM) includes aspects of passive short-term maintenance, such as the visuospatial sketchpad and phonological loop (‘storage buffer’), as well as those of active manipulation (‘central executive’), such as transformation of mental representations. To investigate the neural substrate for WM, previous studies have shown that distributed brain regions exhibit sustained activity during the retention interval of delayed-response tasks (Goldman-Rakic, 1987; Wager & Smith, 2003). In particular, it is proposed that the posterior brain regions play a general role in maintaining the actual contents of representations, as the delay-period activity is correlated with the numbers of objects held in WM (Todd & Marois, 2004; Vogel & Machizawa, 2004). On the other hand, the prefrontal cortex is thought to act as an attentional controller, determining which information will be maintained and updating it in WM, but not participating in the actual maintenance of that information (Cohen *et al.*, 1997; Smith & Jonides, 1999; Rowe *et al.*, 2000; Kawasaki *et al.*, 2006). Thus, early studies provided rich evidence for the existence of brain regions that specialize in each storage buffer and executive function for one certain representation, leading to the hypothesis that the control processes of the WM are achieved by top-down signals from the prefrontal cortex to the posterior regions where the representations are stored (Miller & Cohen, 2001; Curtis & D’Esposito, 2003). Although, thus far, a few neuroimaging studies have approached how the brain regions of executive function are linked to and access the storage systems (Vogel *et al.*, 2005; McNab & Klingberg, 2008), they have not dealt with the dynamic relationships governing brain activity.

To address this issue, it is particularly useful to elucidate the temporal relationships among specific brain regions with regard to their electroencephalographic (EEG) oscillations. It is believed that EEG oscillations reflect the dynamic linking of cell assemblies through synchronization of a large number of neurons underlying a particular function, and that the large-scale synchronization serves to integrate the functions of different cell assemblies (Varela *et al.*, 2001). Previous human scalp-recorded EEG studies have shown modulated theta and alpha rhythms in distributed brain regions and phase synchronization between them during various WM tasks (Jensen & Tesche, 2002; Mizuhara *et al.*, 2004; Sauseng *et al.*, 2005; Kawasaki & Watanabe, 2007; Klimesch *et al.*, 2008). These EEG oscillations might reflect functional links among different processes and different regions. If so, they should have a coordinated structure as spatiotemporal patterns in a functionally relevant way. However, the above studies have not clearly identified the functional coordination of each oscillation in each brain region for different kinds of WM.

The present study aimed to identify the brain oscillatory activity involved in two WM processes (manipulation and maintenance) for two modalities (visual and auditory), and to investigate the dynamic links among them. We used time–frequency (TF) analyses of 62-channel EEG data for two WM manipulation tasks: an auditory WM task, which required mental calculation of numbers presented through an auditory stimulus; and a visual WM task, which required the participants to move a spatial location in a mental representation in accordance with a visual stimulus.

## Materials and methods

### Participants

Fourteen healthy volunteers [10 males and four females; mean age, 27.9 ± 6.8 years (range, 21–41 years); 13 volunteers were right-handed] with normal or corrected-to-normal visual acuity, normal hearing acuity and normal motor performance participated in the EEG experiment. All of the participants gave written informed consent, before the experiments were performed. The study was approved by the Ethical Committee of the RIKEN and was in accordance with the Declaration of Helsinki.

### Auditory working memory condition

**Fig. 1:**
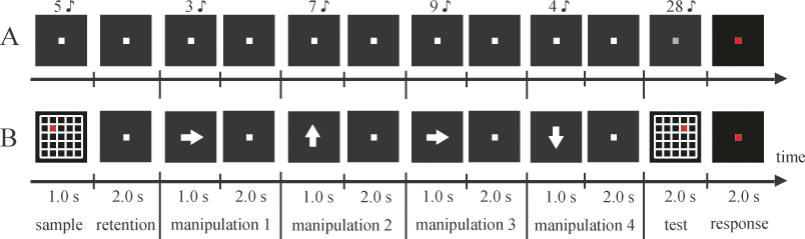
Schematic illustrations of one trial sequence for the auditory (A) and visual (B) working memory (WM) tasks. (A) In the auditory WM task, participants performed a mental calculation for numbers auditorily presented through headphones; the calculation process was repeated four times. (B) In the visual WM task, participants had to memorize the red circle’s position in the sample display and move the circle in their minds, as visually indicated by arrows; they had to perform this sequence of actions four times.

The participants, wearing headphones, faced a computer screen placed 60 cm away. At the beginning of each trial, a word indicating a one-digit number was presented as the auditory stimulus through both right and left headphone speakers within 1 s (sample display, Fig. 1A). The participants were required to memorize the presented number through rehearsal in their minds. After a 2-s retention interval, another one-digit number was presented as the auditory stimulus within 1 s, and the participants were required to add the presented number to the earlier memorized number and memorize the result within 2 s. The participants were required to repeat this mental addition four times, and then to determine whether the total that they had mentally calculated matched the number presented as a probe auditory stimulus after the white fixation point grayed (test display). In half of the trials, a wrong total was presented as the probe stimulus by replacing one of the four presented numbers with a different number. The participants were asked to indicate within 2 s (while the fixation point was red) whether the number was correct by pressing a button. The duration of the inter-trial interval (ITI) was 2 s. The stimulus was generated on a Windows computer, using matlab 7.5.0 (MathWorks, Natick, MA, USA) with the Psychophysics Toolbox extension. The sound of each number was highly distinctive.

### Visual WM condition

At the beginning of each trial, 5 × 5 square grids and a red circle included within one of the squares were presented on the computer screen as the visual stimulus for 1 s (sample display, Fig. 1B). The participants were required to memorize the position of the red circle for 2 s after the visual stimulus disappeared. Subsequently, a white arrow designating the direction towards which they should move the red circle to one grid position in their minds was presented at the center of the screen for 1 s. Participants manipulated the mental representations for 2 s. The arrow was directed upwards, downwards, rightwards, or leftwards. As in the auditory WM condition, they were asked to repeat the mental manipulation four times, and then determine whether the mentally determined position of the red circle that they mentally moved matched a probe visual stimulus (test display). In half of the trials, a wrong probe was presented by changing the direction of the fourth movement from the actual direction. The press of the button, duration of the ITI and creation of the stimulus were identical to those in the auditory WM condition. The sizes of the red circle and the square grids were 1° × 1° and 5° × 5°, respectively.

## Experimental procedure

Each participant completed two separate sessions under auditory WM and visual WM conditions in a counterbalanced order. Each session consisted of 96 manipulations (four manipulations × 24 trials). All participants undertook a behavioral training session before the EEG measurement sessions.

### EEG recordings

EEG recordings were continuously performed using 62 scalp electrodes (Ag/AgCl) embedded in an electrocap, in accordance with the extended version of the international 10/20 system. The sampling rate was 500 Hz. Reference electrodes were placed on the right and left ears. The artefacts related to eye blinks and movements were measured by electrodes placed above and below the left eye for monitoring either eye blinks or vertical eye movements, and by electrodes placed 1 cm from the right and left eyes for monitoring horizontal eye movements. Trials in which the amplitude of any electrode of an EEG epoch exceeded ± 100 μV were rejected from the analysis offline. These EEG data were amplified using NeuroScan equipment (Neuroscan, EI Paso, TX, USA) and filtered in the bandpass range from 0.1 Hz to 50 Hz.

### EEG data pre-processing

We analysed the EEG data for the correct trials. EEG data were segmented into 3-s epochs for the manipulation period from the onset of the instruction for manipulation (e.g. arrow for the visual WM condition). These epochs were subjected to infomax independent components analysis (ICA) using eeglab (Delorme & Makeig, 2004; Institute for Neural Computation, University of California, San Diego, CA, USA) in matlab. The ICA components that were significantly correlated with either the vertical or horizontal electrooculograms were regarded as the components related to eye movement or eye blink; these components were eliminated from the data. The ICA-corrected data were recalculated using regression of the remaining components.

To accurately evaluate the cortical activity under the scalp EEG electrodes without encountering errors caused by volume conduction, we carried out current source density analysis at each electrode position. We applied the spherical Laplace operator to the voltage distribution on the surface of the scalp. This method was performed with the following parameters: the order of the spline, *m* = 4, and the maximum degree of the Legendre polynomial, *n* = 50, with a precision of 10^−5^ (Perrin *et al.*, 1989).

### Wavelet analysis

The TF amplitudes and phases were calculated by wavelet transforms using Morlet’s wavelets with a Gaussian shape in the time domain (standard deviation *σ*_*t*_) and with the frequency domain (standard deviation *σ*_*f*_) located around the center frequency ( *f* ) (Tallon-Baudry *et al.*, 1997). The TF amplitude *E*(*t*, *f* ) for each time point of each trial was the squared norm of the results of convolution of the original EEG signals *s*(*t*) with complex Morlet’s wavelet function *w*(*t*, *f* ): 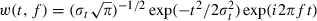

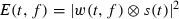
 with *σ*_*f*_ = 1/(2*πσ*_*t*_). The wavelet used was characterized by a constant ratio ( *f*/*σ*_*f*_ = 7), with *f* ranging from 1 Hz to 20 Hz (0.5-Hz steps). The TF amplitude of each event and condition was calculated before or after 1 s of each event epoch to minimize the edge artefact of the low-frequency wavelets, and averaged across single trials. The event-related TF amplitude was calculated by subtracting the baseline data measured in the ITI for each frequency band. For all statistical analyses, a non-parametric Wilcoxon signed-rank test was used across the events or conditions, because the populations of the TF amplitude did not correspond to a Gaussian distribution.

### Within-frequency phase synchronization

To identify the phase relations between any two electrodes, the phase synchronization index (PSI) for each time point and each electrode pair was defined by the equation: 
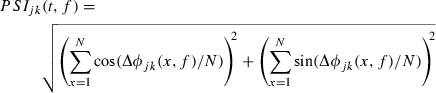
 where Δ*Φ*_*jk*_(*t*, *f* ) is the phase difference between the *j*th and *k*th electrodes, and the number of time points *N* with an interval of 1 s is 500 (Lachaux *et al.*, 1999).

To evaluate the manipulation-related PSI changes, we applied the bootstrap method to the PSIs of each trial of the individual subjects and compared the virtual PSI data obtained during the manipulation periods [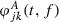] with the baseline data of the ITI period [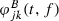]: 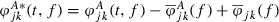

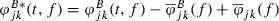
 where 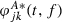
and 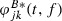 represent the original PSI, and 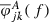, 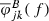 and 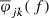 represent the mean 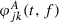, mean 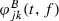, and the average value of all the data, respectively (Mizuhara *et al.*, 2005). Using the non-parametric Wilcoxon signed-rank test for 2000 bootstrapped re-samples of each time point for individual subjects, we calculated the *z*-values at which the PSI values during the manipulation periods were higher than those during the ITI. For the individual subjects’*z*-values, we applied a sign test against the null hypothesis that the *z*-values equal zero.

### Cross-frequency coupling (CFC)

We also calculated the phase–phase cross-frequency coupling (CFC) between the theta (6-Hz) and alpha (12-Hz) oscillations at one electrode. The PSI formula was applied using Δ*Φ*_6 Hz –12 Hz_ (*t*, *j*) as the phase difference between two theta phases (2 × *Φ*_6 Hz_) and the alpha phase (*Φ*_12 Hz_) at the *j*th electrodes, because the relationships between the theta and alpha phases were expressed in the ratio 1 : 2 during both the manipulation and the ITI periods. The CFC was calculated with the equation: 
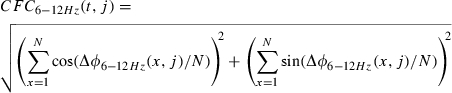

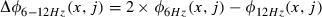


For these data, similar to the case for within-frequency phase synchronization analyses, we compared the PSI between the manipulation and the ITI periods.

## Results

### Behavioral performance

All the participants performed both tasks with high accuracy rates (mean accuracy rate ± standard deviation, 95.2 ± 6.1 and 97.3 ± 4.6 for the auditory and visual WM conditions, respectively). There was no significant difference between the task conditions (Wilcoxon signed-rank test; *Z* = 1.07, *P* = 0.28), indicating that EEG comparison among the different conditions was not influenced by the difficulty of the task.

### Process-specific and modality-specific local synchronizations by oscillatory amplitudes

**Fig. 2:**
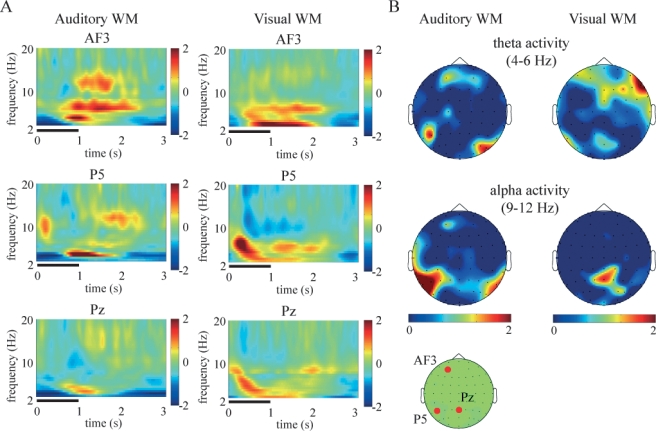
(A) Subject-averaged (*N* = 14) time–frequency amplitudes of the AF3 (top), P5 (middle) and Pz (bottom) electrodes during the manipulation periods under the auditory (left) and visual (right) working memory (WM) tasks. These values, normalized with respect to the inter-trial interval baseline, were averaged across the correct trials of all participants. The thick horizontal lines indicate the presentation of manipulation instructions. (B) Topographic colored scalp maps of the theta (4–6 Hz, top) and alpha (9–12 Hz, middle) amplitudes for 1–2 s from the onset of each manipulation period during the auditory (left) and visual (right) WM tasks. The drawing shows the top view of the scalp. The bottom panel shows the AF3, P5 and Pz electrodes on the recording montage.

To elucidate the spatiotemporal domain, current source density analysis was first applied and wavelet analysis was then performed for the EEG data recorded during the two tasks. The averaged TF amplitudes of the periods in which manipulations were performed four times relative to those of the ITIs showed that the theta amplitudes (4–6 Hz) increased in the frontal and temporal regions under the auditory WM condition and in the frontal and parietal regions under the visual WM condition (Fig. 2A). We selected the AF3, P5 and Pz electrodes as the representative activities of the frontal, temporal and parietal regions, respectively, because of the peak amplitudes associated with these electrodes. The theta amplitudes were sustainably elicited from 1 s to 2 s after the onset of the manipulation cue; furthermore, the theta activity on the AF3 for both conditions, on the P5 for the auditory WM condition and on the Pz for the visual WM conditions was significantly higher than the activity of the ITI (auditory WM: AF3, *Z* = 4.26, *P* < 0.01; P5, *Z* = 2.03, *P* < 0.05; Pz, *Z* = 1.65, *P* = 0.10) (visual WM: AF3, *Z* = 3.53, *P* < 0.01; P5, *Z* = 1.88, *P* = 0.06; Pz, *Z* = 2.04, *P* < 0.05).

The peak frequencies of the frontal enhanced theta amplitudes were approximately 6 Hz during both conditions. On the other hand, enhancement of the alpha amplitudes (9–12 Hz) was mainly observed in the temporal areas under the auditory WM and the parietal areas under the visual WM conditions (auditory WM: AF3, *Z* = 2.98, *P* < 0.01; P5, *Z* = 2.08, *P* < 0.05; Pz, *Z* = 1.81, *P* = 0.07) (visual WM: AF3, *Z* = 1.15, *P* = 0.25; P5, *Z* = 1.35, *P* = 0.17; Pz, *Z* = 2.19, *P* < 0.05). The peak frequencies of the AF3 and P5 alpha amplitudes were approximately 12 Hz under the auditory WM condition, whereas the peak frequency of the Pz alpha amplitude was approximately 9 Hz under the visual WM condition.

To dissociate manipulation-related activity from maintenance activity, we compared the theta and alpha activities during the manipulation periods with those during the retention intervals, which are not directly related to the manipulation periods (Fig. 2B). As a result, the increased P5 alpha activity for the auditory WM and Pz alpha activity for the visual WM also survived during the retention interval, and the amplitudes were not significantly different between the manipulation period and the retention interval (P5 for auditory WM, *Z* = 1.87, *P* = 0.06; Pz for visual WM, *Z* = 0.49, *P* = 0.62). On the other hand, AF3 theta activity was not evident during the retention interval, and the theta amplitudes for manipulation were higher than those for retention in both conditions (AF3 for auditory WM, *Z* = 2.94, *P* < 0.01; AF3 for visual WM, *Z* = 2.18, *P* < 0.01). Thus, the scalp localization of these activities is well distinguished under distinct conditions. The results suggest that the frontal theta activity was associated with executive functions, and that the parietal and temporal alpha activities were involved in the storage buffers and manipulation of the visual and auditory WMs, respectively. This gives rise to the following questions: (i) can inter-regional linking be found in EEG rhythms, and (ii) how does control of the storage buffer during the manipulation period appear in the EEG?

### Regional linking elucidated by phase synchronizations

**Fig. 3:**
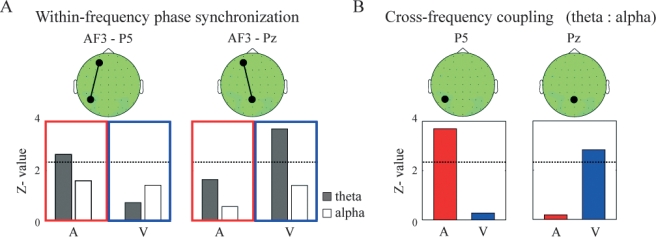
(A) The *z*-values of the within-frequency phase synchronization index (PSI) between AF3 and P5 (left) and between AF3 and Pz (right) during the manipulation periods in comparison with the ITI under the auditory (A) and visual (V) working memory (WM) conditions. The gray and white bars indicate the *z*-values of the theta and alpha synchronizations, respectively. (B) The *z*-Values of the cross-frequency PSI between the theta and alpha phases at P5 (left) and Pz (right) for the manipulation periods in comparison with the ITI under the auditory (A) and visual (V) WM conditions. The dotted lines in each panel denote the threshold value (*P* < 0.01).

To further investigate the interaction among the above regions, we calculated the PSI between a pair of representative electrodes at the theta (6 Hz) and alpha (12 Hz) bands. The bootstrapped results revealed that the theta (6 Hz) PSI_AF3–P5_ in the auditory WM and the theta PSI_AF3–Pz_ in the visual WM increased significantly during the manipulation periods (for 1 s after offset of the manipulation cue) as compared with the corresponding values during the ITI (*P* < 0.01; Fig. 3A). Significant increases in the theta PSI_AF3–P5_ for the visual WM and the PSI_AF3–Pz_ for the auditory WM were not found. In contrast, the alpha (12 Hz) PSI_AF3–P5_ and the PSI_AF3–Pz_ during the auditory and visual WM conditions were not significantly different between manipulation periods and the ITI. The PSI results indicate that the frontal regions dynamically link with the task-relevant posterior region through theta synchronization during the manipulation period.

### Theta–alpha coupling in manipulation periods

The present results show that the storage buffer is characterized by alpha oscillations and that the manipulation period is associated with concurrent theta and alpha oscillations. Control of the storage buffer during the manipulation period might be observed in any coupling between theta and alpha oscillations. We examined the WM manipulation in each brain region by analysing the phase–phase CFC (O’Keefe & Dostrovsky, 1971; Palva *et al.*, 2005). The 6-Hz phase and 12-Hz phase at the single electrodes exhibited a 1 : 2 ratio relationship during both the manipulation and the ITI periods. Therefore, we calculated the PSI between two 6-Hz phases and the 12-Hz phases at the P5 and Pz electrodes. (It should be noted that this 1 : 2 frequency coupling is not singular at the 6-Hz and 12-Hz components, but it is robust between the theta-band and alpha-band components.) The PSI_6 Hz – 12 Hz_ values of the P5 and Pz electrodes were significantly higher during the manipulation periods of only the relevant WM condition (P5 for the auditory WM condition and Pz for the visual WM condition) than at the ITI (*P* < 0.01; Fig. 3B). These CFCs may suggest that different operations encoded in different frequency bands are dynamically integrated at each brain region during the manipulation period.

Also, CFCs might be able to appear because of an artefact of another oscillation component around 8 Hz that could be superposed on both the 6-Hz and 12-Hz components in wavelet analyses. In order to avoid this overlap in the Fourier domain, we additionally conducted the cross-frequency analyses by using short Morlets ( *f*/*σ*_*f*_ = 12). Under the shorter Morlet conditions, the 6-Hz and 12-Hz CFCs were also observed in the P5 and Pz electrodes for only the relevant WM conditions. This result excludes the artefactual appearance of the CFCs. In summary, these CFCs may suggest that different operations encoded in different frequency bands are dynamically integrated at each brain region during the manipulation period.

## Discussion

Our results for the oscillatory amplitudes demonstrated a clear dissociation within a distributed WM network between two functions, namely, the central executive and storage buffer functions. Frontal theta activity was mainly observed in the manipulation period, and not in the maintenance periods. On the other hand, posterior alpha activity was enhanced both in the manipulation period and and in the maintenance period. The posterior regions were divided, according to the WM modality, into the temporal areas (P5 electrode) for the auditory WM and the parietal areas (Pz electrode) for the visual WM. These results support the idea that the frontal cortex is associated with active manipulation and the posterior regions are involved in simple maintenance of each modality (Postle *et al.*, 1999; Smith & Jonides, 1999; Rowe *et al.*, 2000; Curtis & D’Esposito, 2003; Veltman *et al.*, 2003; Wager & Smith, 2003). In addition, these results clarify the roles of both oscillations, which reflect the different local synchronizations within specific cell assemblies, in the WM process: theta for manipulation, and alpha for maintenance. It is worth noting that the retention interval of our tasks might include not only maintenance of representations but also other functions, such as preparation, initialization of the representations, and maintenance of task rules. Therefore, the alpha frequencies of the retention intervals and those of the manipulation periods were slightly different. Although the functional roles of theta and alpha amplitudes in the WM are not clearly understood (Klimesch *et al.*, 2008), it is possible that, in some of these studies, the use of complex WM tasks led to contamination of the manipulation and maintenance processes, such as *n*-back tasks, which can also be interpreted as selective maintenance in the WM of sequentially represented items to establish ‘temporally lined-up’ representations (Jensen & Tesche, 2002; Jensen *et al.*, 2002 ).

With regard to how such frontal and posterior WM-related systems are functionally related, the current study showed that, in comparison with the case during the ITI, the within-frequency theta-phase synchronizations between the frontal and posterior regions increased during the manipulation periods. Interestingly, theta-phase synchronizations were observed only in the frontal and relevant cortical areas where the representations are stored; that is, frontal–temporal synchronization was observed in the auditory WM, and frontal–parietal synchronization in the visual WM. Several studies have also reported increased theta-phase synchronization during several WM tasks (Sauseng *et al.*, 2005; Mizuhara & Yamaguchi, 2007). Together with previous evidence, our findings can be interpreted as revealing that long-range theta synchronizations connect the central executive functions to task-relevant information in the storage buffers. In contrast to what was found for theta rhythms, alpha-phase synchronizations were not observed during either the auditory or the visual WM tasks, which indicates that global alpha synchronization is not directly involved in executive functions. Indeed, previous studies have shown alpha-phase synchronizations during sensory–semantic encoding, retrieval, or sensory–motor tasks, rather than executive functions, and these synchronizations have mainly been found in the local areas (i.e. between adjacent electrodes) (Sauseng *et al.*, 2005; Klimesch *et al.*, 2008).

Finally, this study showed CFC between two theta phases (6 Hz) and the alpha phase (12 Hz) at the frontal and task-relevant posterior electrode (temporal for the auditory WM, and parietal for the visual WM) during the manipulation periods. This result clarifies the integrative brain structures that facilitate collaboration between different functional oscillations in order to accomplish a given cognitive function. Previous studies emphasized that CFC reflected the interactions between different cell assemblies operating in different oscillations; for example, theta–gamma coupling on the frontal electrodes during the retention interval of a short-term memory task was proposed to reflect functional linkage between the frontal and cortico-limbic networks (Burgess & Ali, 2002; Schack *et al.*, 2002; Klimesch *et al.*, 2008). This evidence might lead to the interpretation that long-range theta synchronizations induce the gating of relevant information in the storage buffer, as alpha oscillations have also been understood to be associated with the gating of sensory inputs (Worden *et al.*, 2000; Payne & Kounios, 2009).

Our findings in relation to several brain oscillations might be closely linked to previous psychological research on limited WM capacity. The characteristics of the storage-related alpha oscillations (e.g. peak areas and frequencies) are different between the auditory and visual WMs, a result that is consistent with suggestions offered by previous psychological studies that simple storage capacities for different modalities are independent and not influenced by each other (Scarborough, 1972; Luck & Vogel, 1997). On the other hand, overlapped theta synchronizations for executive functions, regardless of the modalities involved, might lead to dual-task interference, that is, degraded performance when performing two tasks simultaneously relative to a single task (e.g. psychological refractory period) (Pashler, 1994; Logan & Gordon, 2001). Indeed, prefrontal cortex activity increased in the dual task, so that the shortened stimulus onset asynchrony between the two tasks would then be the neural factor responsible for the bottleneck of executive functions (Herath *et al.*, 2001; Jiang *et al.*, 2004; Marois & Ivanoff, 2005). In addition, future studies on dual tasks should clarify the TF characteristics of brain activity, and investigate how our mind can switch between tasks and how irrelevant information is stored during manipulation of the other relevant information.

**Fig. 4:**
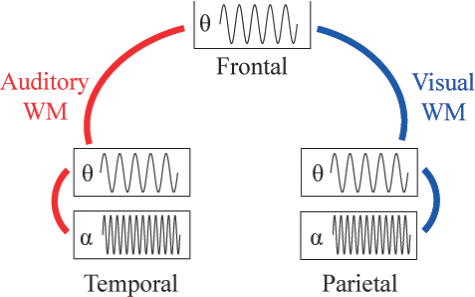
Schematic illustrations of working memory (WM) manipulation of the auditory and visual representations. The external information is stored in the modality-specific posterior region by the alpha rhythms. For manipulation of the stored representations, the theta synchronizations connect the frontal central executive regions with the posterior regions where the relevant information is stored. Moreover, the theta and alpha oscillations interact at the relevant posterior regions.

In summary, our findings on local synchronizations, within-frequency regional synchronizations and CFC of the theta and alpha rhythms demonstrate that dynamic linkage between theta oscillations and modality-specific alpha oscillations mediates communication between the central executive functions and storage buffer functions in WM (Fig. 4). Human EEG evidence of theta and alpha activity suggests a possible strategy for a hierarchical control structure for the multiple operations in different spatiotemporal domains within the cortical regions for several cognitive processes, as well as in WM. Concurrent theta and alpha activity may contribute to a dual gating mechanism for neural firing, the verification of which is beyond the scope of this study.

## Abbreviations

CFC: cross-frequency coupling
EEG: electroencephalographic
ICA: independent components analysis
ITI: inter-trial interval
PSI: phase synchronization index
TF: time–frequency
WM: working memory
